# GMMA Is a Versatile Platform to Design Effective Multivalent Combination Vaccines

**DOI:** 10.3390/vaccines8030540

**Published:** 2020-09-17

**Authors:** Francesca Micoli, Renzo Alfini, Roberta Di Benedetto, Francesca Necchi, Fabiola Schiavo, Francesca Mancini, Martina Carducci, Elena Palmieri, Cristiana Balocchi, Gianmarco Gasperini, Brunella Brunelli, Paolo Costantino, Roberto Adamo, Diego Piccioli, Allan Saul

**Affiliations:** 1 GSK Vaccines Institute for Global Health (GVGH) S.r.l., 53100 Siena, Italy; renzo.x.alfini@gsk.com (R.A.); roberta.x.di-benedetto@gsk.com (R.D.B.); francesca.x.necchi@gsk.com (F.N.); fabiola.schiavo@yahoo.it (F.S.); francesca.x.mancini@gsk.com (F.M.); martina.x.carducci@gsk.com (M.C.); elena.x.palmieri@gsk.com (E.P.); gianmarco.x.gasperini@gsk.com (G.G.); allan.saul@honorary.burnet.edu.au (A.S.); 2 GSK, 53100 Siena, Italy; cristiana.x.balocchi@gsk.com (C.B.); brunella.x.brunelli@gsk.com (B.B.); paolocostantino1@gmail.com (P.C.); roberto.x.adamo@gsk.com (R.A.); diego.x.piccioli@gsk.com (D.P.)

**Keywords:** GMMA, Outer Membrane Vesicles (OMV), glycoconjugate, vaccine

## Abstract

Technology platforms are an important strategy to facilitate the design, development and implementation of vaccines to combat high-burden diseases that are still a threat for human populations, especially in low- and middle-income countries, and to address the increasing number and global distribution of pathogens resistant to antimicrobial drugs. Generalized Modules for Membrane Antigens (GMMA), outer membrane vesicles derived from engineered Gram-negative bacteria, represent an attractive technology to design affordable vaccines. Here, we show that GMMA, decorated with heterologous polysaccharide or protein antigens, leads to a strong and effective antigen-specific humoral immune response in mice. Importantly, GMMA promote enhanced immunogenicity compared to traditional formulations (e.g., recombinant proteins and glycoconjugate vaccines), without negative impact to the anti-GMMA immune response. Our findings support the use of GMMA as a “plug and play” technology for the development of effective combination vaccines targeting different bugs at the same time.

## 1. Introduction

GMMA (Generalized Modules for Membrane Antigens) is a technology platform with great potential for affordable and effective vaccines [1]. Gram-negative bacteria spontaneously release blebs of the outer membrane, also called Outer Membrane Vesicles (OMV), that have been considered attractive for vaccine design [2,3]. OMV, in fact, are basically components of Gram-negative bacteria containing surface-exposed antigens in native conformation and orientation, together with immunostimulatory molecules, such as lipopolysaccharide (LPS), lipoproteins or peptidoglycans. GMMA are OMV derived from bacteria genetically engineered to enhance OMV release [4,5]. Mutations are also introduced to modify the lipid A structure of LPS to minimize the capacity of GMMA to promote reactogenic response once injected, still maintaining an immunopotentiator effect of the Toll-like receptor 4, which is triggered by lipid A [6,7]. GMMA can be produced at high yields using a simple and robust process of manufacturing, potentially leading to affordable vaccines [1,4]. An advanced GMMA-based vaccine to prevent *Shigella sonnei* infection has been shown to be well-tolerated and immunogenic in clinical trials in healthy adults and endogenous population [8,9,10].

Conjugation of antigens to appropriate carrier proteins is an established procedure for improving immunogenicity, particularly for polysaccharides [11,12]. Bacterial capsular polysaccharides are T-cell-independent antigens which, when delivered alone, give rise to an immune response lacking several important properties, such as immunological memory, affinity maturation, persistence of antibody response and ability to induce adequate protection in infants and children under 2 years of age. Conjugation to a carrier protein provides saccharide antigens with a T-cell-dependent response, resulting in an improved germinal centers’ formation, which leads to immunological memory, isotype switching and affinity maturation of B cell receptors. Consequently, vaccination generates enhanced immunogenicity and protective efficacy, especially in infants [13,14]. 

Currently, there are several diseases that are a serious threat to mankind for which vaccines are not available, and the development of which is often restricted by a lack of commercial sustainability [15]. Recently, the increase of antimicrobial resistance has created an additional serious global problem [16,17]. Thus, research and development for new or improved vaccines together with the efforts to accelerate their market release are considered by the World Health Organization (WHO) as part of a strategic approach to prevent diseases globally [18]. From this point of view, the development of new technologies to facilitate vaccine design is recommended.

Here, we have tested GMMA as a carrier for protein and polysaccharide antigens. Chemical conjugation is a straightforward tool to decorate GMMA with antigens from pathogens different from those from which the GMMA are derived. Our primary goal was to investigate if conjugation to GMMA increases immunogenicity in comparison to its unconjugated counterpart, or, in the case of polysaccharides, results in immunogens that are at least as immunogenic as a conventional conjugate. We also demonstrate that multiple antigens can be simultaneously presented on the same GMMA particle with no immune interference, supporting the use of the GMMA platform as a “plug and play” technology for the development of effective multi-functional antigens targeting different bugs at the same time. 

## 2. Materials and Methods 

### 2.1. Source of GMMA and Antigens

*S*. Typhimurium GMMA (obtained from isolate 1418 Δ*tolR* mutant strain), *S. sonnei* GMMA (obtained from Δ*tolR* Δ*virG* Δ*htrB* 53G mutant strain) and *Neisseria meningitidis* serogroup B (MenB) GMMA (produced from a Δ*synX*, Δ*ctra*, Δ*gna33*, Δ*lpxL1 Neisseria meningitidis* mutant strain) were produced and characterized as previously described [4,19]. *Plasmodium falciparum* circumsporozoite protein (CSP) and *P. falciparum* Pfs25 recombinant proteins (42.5 and 18 kDa, respectively) were kindly provided by the Malaria Vaccine Initiative (PATH, Seattle, WA, USA) and the Laboratory of Malaria Immunology and Vaccinology (HHS/NIH/NIAID, Bathesda, MD, USA), respectively. *Escherichia coli* SslE, factor adherence *E. coli* (FdEc), *Neisseria meningitidis* factor H binding protein variant 1 (fHbp v1) recombinant proteins (175, 41.7 and 27 kDa respectively), *Hemophilus influenzae* type b (Hib) and *Neisseria meningitidis* serogroups A and C (MenA and MenC) oligosaccharides were provided by GSK Vaccines. 

### 2.2. Synthesis and Characterization of the GMMA Conjugates

Conjugates were synthesized as described below. The main characteristics of all the conjugates tested in this study are reported in Table S1.

#### 2.2.1. Linkage of Heterologous Saccharides to GMMA

##### Conjugation via SH-Maleimido Chemistry

*S*. Typhimurium GMMA derivatization with -SH linker (N-acetyl-DL-homocysteine thiolactone): GMMA were resuspended at 4 mg/mL in 100 mM borate buffer, pH 8, and added to equal volume of activation buffer containing 2.6 mg/mL Dithiothreitol (DTT), 13.16 mg/mL Ethylenediaminetetracetic acid (EDTA) and 7.04 mg/mL N-acetyl-DL-homocysteine thiolactone in 100 mM borate buffer, pH 11, for a 7-fold ratio of thiolactone to NH_2_ groups on GMMA. The reaction was mixed at room temperature for 4 h, GMMA-SH were then purified by ultracentrifugation (110,000 rpm, 4 °C, 1 h) and resuspended in 100 mM 2-(*N*-morpholino)ethanesulfonic acid (MES) buffer, pH 6 (for conjugation to CSP and fHbp), or pH 5 (for conjugation to Pfs25). GMMA-SH were characterized by micro Bicinchoninic acid (BCA) protein assay (Pearce/ThermoFisher) (80–90% protein recovery) and 2,4,6-Trinitrobenzenesulfonic acid (TNBS) [20], showing that 40% of NH_2_ groups were activated.

CSP derivatization with EMCS (N-ε-malemidocaproyl-oxysuccinimide ester) linker: CSP at the concentration of 270 µg/mL in phosphate buffered saline (PBS) was added to EMCS linker (as a 10 mg/mL solution in DMSO) to have a 1:1 molar ratio of linker to Lys residues of the protein. The solution was mixed at room temperature for 4 h and then the derivatized protein was purified by PD10 desalting column (GE Healthcare Life Sciences, Chicago, IL, USA) against MES 10 mM, pH 6. The resulting product was characterized by micro BCA (64% recovery) and Sodium Dodecyl Sulphate-Poly-Acrylamide Gel Electrophoresis (SDS-PAGE) to verify that there were no covalently bound aggregates.

Pfs25 derivatization with EMCS linker: EMCS linker as a 10 mg/mL solution in Dimethyl Sulfoxide (DMSO) was added to Pfs25 at 3.0 mg/mL in PBS to target 30% of Lys residues of the protein. The solution was mixed at room temperature for 4 h and then the derivatized protein was purified by chromatography on a PD10 column equilibrated with MES 10 mM, pH 5. The resulting product was characterized by micro BCA (100% recovery) and SDS-PAGE to verify no formation of aggregates. Analysis by Matrix-Assisted Laser Desorption/Ionization-Mass Spectrometry (MALDI-MS) confirmed the introduction of an average number of 7–8 linkers per protein.

fHbp v1.1 derivatization with EMCS linker: EMCS linker as a 10 mg/mL solution in DMSO was added to 1.25 mg/mL of fHbp in PBS to have a 0.2:1 molar ratio of linker to Lys residues of the protein. The solution was mixed at room temperature for 4.5 h and then the derivatized protein was purified by chromatography on a PD10 column equilibrated with MES 10 mM, pH 6. The resulting product was characterized by micro BCA (95% recovery), High-Performance Liquid Chromatography-Size Exclusion Chromatography (HPLC-SEC) and SDS-PAGE, showing no protein aggregation, and MALDI-MS, indicating an average of three linkers introduced per molecule of protein.

Conjugations: Each protein, previously derivatized with EMCS linker, was conjugated to GMMA-SH. Conjugation was performed in 100 mM MES, pH 5 or 6, according to the protein used. The reaction was mixed at room temperature for 4–5 h, and the conjugate was purified by ultracentrifuge (110,000 rpm, 4 °C, 1 h) and resuspended in PBS. GMMA concentration and ratio of protein antigen to GMMA in the conjugation mixture were changed according to the protein linked and are detailed in Table S1. 

##### Conjugation via Reductive Amination Chemistry

GMMA oxidation: *S. sonnei* GMMA were oxidized at a concentration of 2.1 mg/mL with NaIO4 5 mM for 30 min at a 25 °C controlled temperature, in the dark. Excess NaIO_4_ was quenched with Na_2_SO_3_ at a final concentration of 10 mM, for 15 min at room temperature. Oxidized GMMA were characterized by High-Performance Anion-Exchange Chromatography-Pulsed Amperometric Detection (HPAEC-PAD) and had 33% sugar units oxidized.

SslE (*w*/*w* ratio of GMMA to SslE 1:1 at a GMMA concentration of 1.23 mg/mL) was directly added to quenched *S. sonnei*-oxidized GMMA, in 100 mM NaPi, pH 6.5, with NaBH_3_CN (1:1 *w*/*w* ratio with GMMA). After gently mixing overnight at room temperature, the conjugate was purified by ultracentrifuge (110,000 rpm 4 °C, 1 h) and resuspended in PBS. 

##### Conjugation through BS3 Chemistry

GMMA activation with BS3 linker: *S. sonnei* GMMA, at a protein concentration of 4.0 mg/mL in 100 mM borate buffer, pH 9, was added to BS3 linker at a final concentration of 50 mg/mL in the reaction mixture. The mixture was incubated at 25 °C for 30 min, then activated GMMA were purified by ultracentrifugation (110,000 rpm, 16 min, 4 °C). Resulting GMMA (70% recovery by micro BCA) had 43.8% of NH_2_ groups derivatized with the BS3 linker, according to the TNBS colorimetric method [20].

Purified activated GMMA were added to FdeC in PBS buffer with a *w*/*w* ratio of GMMA to protein antigen of 1:1 and a GMMA concentration of 5.75 mg/mL. After gently mixing overnight at room temperature, the conjugate was purified by ultracentrifuge (110,000 rpm, 4 °C, 1 h) and resuspended in PBS. 

##### Synthesis of the Bivalent Conjugate

Purified GMMA activated with the BS3 linker were added to FdeC protein antigen in PBS buffer with a *w*/*w* ratio of GMMA to FdeC of 1:1 and a GMMA concentration of 6.45 mg/mL. After gently mixing overnight at room temperature, the conjugate (*S. sonnei* GMMA BS3-FdeC) was purified by ultracentrifuge (110,000 rpm, 4 °C, 1 h) and resuspended in NaPi 100 mM, pH 6.5, for a further conjugation step. After having verified conjugate formation by SDS-PAGE/Western blot, the *S. sonnei* GMMA BS3-FdeC conjugate at the concentration of 2.1 mg/mL was incubated with NaIO_4_ 5 mM for 30 min at a 25 °C controlled temperature, in the dark. Excess of NaIO_4_ was quenched with Na_2_SO_3_ at a final concentration of 10 mM, for 15 min at room temperature. SslE protein antigen (*w*/*w* ratio of GMMA conjugate to SslE 1:1 at a GMMA concentration of 1.18 mg/mL) and NaBH_3_CN (1:1 *w*/*w* ratio with GMMA) were directly added to the reaction mixture. After gently mixing overnight at room temperature, the conjugate ((*S. sonnei* GMMA-BS3-FdeC)ox-SslE) was purified by ultracentrifuge (110,000 rpm, 4° C, 1 h) and resuspended in PBS. 

#### 2.2.2. Linkage of Heterologous Saccharides to GMMA

##### Conjugation via SIDEA (Adipic Acid Bis(N-hydroxysuccinimmide)) Chemistry

MenA, MenC or Hib oligosaccharides terminally activated with SIDEA as previously described [21] were added to a suspension of GMMA in NaPi 50 mM, pH 7.2. The mixture was stirred overnight at room temperature. Different conjugation conditions were used according to the polysaccharide linked and the GMMA used, as detailed in Table S1. Conjugates were purified by ultracentrifugation (110,000 rpm, 4 °C, 1 h) and recovered in PBS. For the synthesis of the bivalent conjugate, the same procedure was used by simultaneously adding MenA and MenC oligosaccharides to GMMA.

### 2.3. Conjugate Characterization

Conjugates were characterized by micro BCA for total protein recovery and SDS-PAGE/Western blot analysis to confirm conjugate formation (Figure S1). To quantify the amount of linked protein antigen, amino acid quantification was used. Amount of saccharide antigen linked was determined by HPAEC-PAD after performing acid hydrolysis directly on GMMA, as previously described [22,23,24,25,26,27]. It was verified that there was no interference from GMMA in the quantification of each saccharide. Nanoparticle Tracking Analysis (NTA) was used to count the number of GMMA particles in solution [19] and estimate the number of polysaccharide chains per GMMA (Table S1).

Absence of free proteins was estimated by analysis of the purified conjugates by HPLC-SEC by using a TSK gel 4000–6000 PW columns (Tosoh Bioscience LCC, King of Prussia, PA, USA) in series and eluting with PBS for 60 min at a flow rate of 0.5 mL/min [19]. Percentage of free saccharide was calculated by solid phase extraction (SPE) using a C4 cartridge (BGB Analytik, Böckten, Switzerland) followed by HPAEC-PAD analysis [25,26].

A detailed characterization of all GMMA conjugates tested in this study is reported in Table S1.

### 2.4. Amino Acid Analysis

Gas phase hydrolysis of the samples (GMMA conjugates and corresponding GMMA alone and unconjugated protein antigen) was performed. All samples were prepared in triplicate, as follows: a volume corresponding to 20 μg total protein as estimated by micro BCA was pipetted into a glass test tube (Waters, Milford, CT, USA) and evaporated to dryness using a centrifugal evaporator (ThermoFisher Scientific, Waltham, MA, USA). Once dried, samples were placed inside the hydrolysis vessel and 200 μL of a 6 M HCl solution containing 0.1% (*w*/*v*) phenol was added inside the vessel outside the sample tubes. After performing 3–4 alternating vacuum-nitrogen flushing steps to remove oxygen from the hydrolysis vessel, the sealed vessel was placed in an oven at 114 °C for the required time (usually 16, 24 or 48 h). Then, sample tubes were dried under vacuum in a SpeedVac.

Hydrolyzed samples were resuspended in 100 μL of 100 mM HCl, vortexed, then 10 μL were derivatized with 6-aminoquinolyl-N-hydroxysuccinimidyl carbamate (AQC) and analyzed by Ultra-Performance Liquid Chromatography (UPLC) following Waters AccQTag Ultra kit instructions. A calibration curve (in the range 15–250 nmol/mL for each amino acid) was built by diluting the Amino Acid Hydrolysate Standard (2.5 mM) from the Waters kit with Milli-Q water. The A260 of the derivatized amino acids were measured with a Photometric Diode Array (PDA) detector. Empower 3 (Waters) software was used for system control and data acquisition.

The proportion of amino acids in the conjugate coming from the antigen (*β_a_*) and from the GMMA (*β_G_*) was determined by finding the values of *β_a_* and *β_G_* that minimized the sum of errors squared (Equation (1)).

(1) $$SSE=\overset{15}{\underset{i=1}{\sum }}{\left(\alpha_{ci}-\beta_{a}\alpha_{ai}-\beta_{G}\alpha_{Gi}\right)}^{2}$$

where *α_ci_* is the proportion of the *i*th amino acid in the conjugate hydrolysate, *α_ai_* is the proportion of the *i*th amino acid in the antigen hydrolysate and *α_Gi_* is the proportion of the *i*th amino acid in the GMMA hydrolysate, and this is summed over 15 amino acids (20 natural amino acids excluding Asn and Gln that are converted to Asp and Glu respectively, and Trp, Cys and Met, that are totally or partially destroyed during hydrolysis). Note that *β_a_* + *β_G_* = 1.

### 2.5. SDS-PAGE/Western Blot

GMMA conjugates were analyzed by SDS-PAGE/Western blot in comparison to free protein antigens and GMMA to verify conjugate formation. Samples (5–20 µL, with a protein content of 2–10 µg) were mixed with NuPAGE SDS sample buffer (1/5 *v*/*v*) and loaded on the gel (3–8% or 7% Tris-acetate NuPAGE, Invitrogen, Carlsbad, CA, USA). The gel was electrophoresed at 45 mA in NuPAGE Tris-Acetate SDS running buffer (20x, Invitrogen). The gel was trans blotted (iBlot, Invitrogen) to nitrocellulose membrane (Invitrogen). The membrane was blocked with 3% BSA-PBS Tween-20 0.05% and immuno-stained with primary antibody (2 h at room temperature, RT) followed by anti-mouse or anti-rabbit IgG (1 h at RT) conjugated to alkaline phosphatase or to Horseradish Peroxidase respectively, and developed with the alkaline phosphatase substrate kit (Sigma-Aldrich, St. Louis, MO, USA) or Opti-4CN (Bio-Rad, Hercules, CA, USA). Both primary and secondary antibodies were diluted in 0.1% BSA-PBS with 0.05% Tween-20 (BSA-PBS Tween buffer).

### 2.6. Immunogenicity Studies in Mice and Rats

All animal sera were used in this study derived from immunization experiments performed at the GSK Animal Facility in Siena or at Toscana Life Sciences Animal Facility (Siena, Italy), in compliance with the relevant guidelines (Italian D. Lgs. n. 26/14 and European directive 2010/63/UE) and the institutional policies of GSK. The animal protocols were approved by the Animal Welfare Body of GSK Vaccines, Siena, Italy, the Italian Ministry of Health and Animal Welfare Body of Toscana Life Sciences (Approval number 804/2015-PR), and the Italian Ministry of Health (Approval number 479/2017-PR). 

CD1 5-week-old outbred female mice were immunized subcutaneously (s.c.) or intramuscularly (i.m.) at days 0 and 28. Adult rats were immunized i.m. at days 0 and 28. For all formulations with aluminum hydroxide (Alhydrogel), it was verified that the antigens (GMMA, free antigens and conjugates) were almost completely adsorbed (<10% free antigens as verified by SDS-PAGE silver staining). 

Anti-antigen-specific IgG levels were measured at days 1, 27 and 42 by enzyme-linked immunosorbent assay (ELISA) [28]. Purified CSP, Pfs25, fHbp v1, SslE and FdeC proteins were used for ELISA plate-coating at 1 µg/mL in carbonate buffer or PBS buffer (the latter for SslE and FdeC). Purified O-antigen (OAg) from *S*. Typhimurium was used for ELISA plate-coating at 5 µg/mL in carbonate buffer, purified MenA and MenC capsular polysaccharides were used at 5 µg/mL in PBS pH 8.2, purified Shigella sonnei LPS was used at 0.5 µg/mL in PBS and Hib polysaccharide conjugated to Human Serum Albumin (HSA) was used at 2 µg/mL in PBS. ELISA units were expressed relative to a mouse antigen-specific antibody standard serum curve, with the best five-parameter fit determined by a modified Hill plot. One ELISA unit is defined as the reciprocal of the dilution of the standard serum that gives an absorbance value equal to 1 in this assay. Each mouse serum was run in triplicate.

Serum Bactericidal Activity (SBA) against meningococcal (MenA, MenC and MenB) and *S*. Typhimurium strains was tested using baby rabbit complement, as previously described [21,29,30].

### 2.7. Statistical Analysis

Datasets were analyzed using a two-tailed nonparametric Mann–Whitney test with Prism (GraphPad Software, San Diego, CA, USA). *p*-values lower than 0.05 were considered statistically significant, and *p*-values were rounded to the nearest larger number.

## 3. Results

### 3.1. GMMA as a Carrier for Protein Antigens

We started by testing the concept of GMMA as a carrier for protein antigens. We used *P. falciparum* Pfs25 and CSP as model antigens and displayed them on *S.* Typhimurium GMMA, with the potential to cover both invasive nontyphoidal salmonellosis and malaria. Since both diseases affect mainly young children in Africa [31,32,33], there is a rationale for a combination vaccine.

To determine whether conjugation to GMMA led to an increased immunogenicity of the protein antigens, we compared the humoral immune response of mice immunized with protein antigen-GMMA conjugates with corresponding recombinant proteins alone or physically mixed with GMMA, at the same doses of antigen and GMMA. 

**Figure 1:**
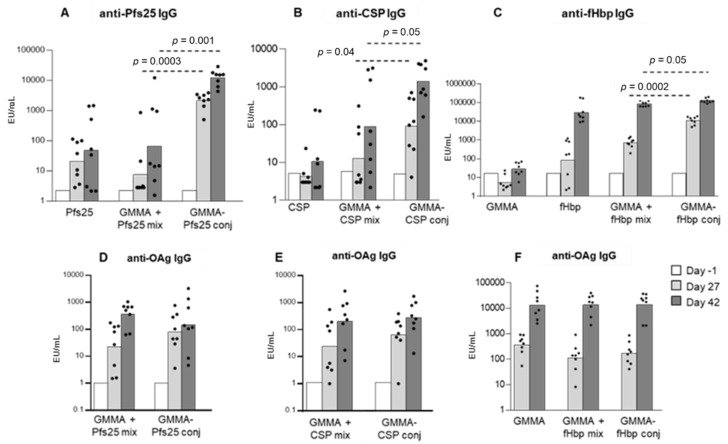
GMMA as a carrier for protein antigens: antigen-specific IgG response. Eight CD1 mice per group were s.c. immunized at days 0 and 28, with *S.* Typhimurium GMMA conjugates of Pfs25 (0.1 µg Pfs25/dose), CSP (0.1 µg CSP/dose) or fHbp (0.75 µg fHbp/dose). Protein antigens, GMMA alone or their physical mixtures were used as controls. Formulations were tested with Alhydrogel. Sera were collected at days 1, 27 and 42 and analyzed for anti-protein antigen-specific IgG response (**A**–**C**) and anti-OAg IgG response (**D**,**E**). Summary graphs of anti-antigen-specific IgG geometric mean units (bars) and individual antibody levels (dots) are reported. In graphs (**D**–**F**), no significant differences in ELISA units were observed. Datasets were analyzed using a two-tailed nonparametric Mann–Whitney test. *p*-values (rounded to the nearest larger number) less than 0.05 were considered statistically significant.

We observed that at low immunogenic doses of Pfs25 or CSP antigens [34,35,36,37], the conjugation to *S*. Typhimurium GMMA strongly enhanced the anti-antigen-specific IgG response compared not only to the antigen alone but also to the antigen physically mixed to GMMA (Figure 1A,B). The enhancement of the antigen-specific antibody response was observed after just one immunization. Importantly, we also observed that the presence of the malaria protein antigens on GMMA surface did not impact on the serum IgG response to Salmonella OAg, considered the major protective antigen for this pathogen (Figure 1D,E). 

Subsequently, we immunized mice with MenB fHbp chemically conjugated to *S*. Typhimurium GMMA or with the corresponding physical mixture of fHbp and GMMA at the same doses or GMMA and fHbp alone. There is a clinical rationale in developing a vaccine that targets both nontyphoidal *Salmonella* and *Neisseria meningitidis*, both prevalent in Sub-Saharan Africa [38,39,40]. This *Neisseria meningitidis* antigen allowed us to measure not only the antigen-specific IgG production, but, also the functionality of the antigen-specific antibody response. For *Neisseria meningitis*, SBA is considered a correlate of protection in humans and consequently, it is an optimal functional assay to generate a proof of concept of the effectiveness of the humoral immune response in mice [41,42]. 

**Figure 2:**
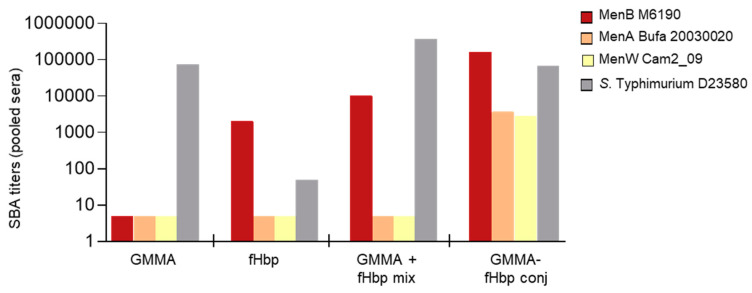
GMMA as a carrier for protein antigens: functionality of the antibody response. Eight CD1 mice per group were s.c. immunized at days 0 and 28, with *S.* Typhimurium GMMA conjugate of fHbp (0.75 µg fHbp/dose). Protein antigen, GMMA alone or their physical mixture were used as controls. Formulations were tested with Alhydrogel. SBA titers of pooled sera collected at day 42 from each group against a panel of meningococcal strains and the *S.* Typhimurium strain are reported.

The GMMA conjugate strongly enhanced the anti-fHbp IgG response compared to the physical mixture or the antigen alone after one immunization, but not after the second (Figure 1C). Although the magnitude of the responses after the second immunization was similar for the fHbp-GMMA conjugate and for fHbp, there was a marked difference in the quality of the response as judged by two criteria: (1) The SBA elicited when tested on MenB with an homologous sequence of fHbp was substantially higher with fHbp-GMMA than with unconjugated fHbp alone or mixed with GMMA (Figure 2), and (2) the specificity of the response: fHbp-GMMA, but not unconjugated fHbp alone or the mixture of fHbp and GMMA, elicited a broad specificity, reacting strongly with MenA and MenW strains with heterologous fHbp sequences (Figure 2).

Importantly, the functionality of the sera, as measured by SBA using the *S*. Typhimurium invasive Malawian clinical isolate D23580, was not decreased by chemical conjugation of fHbp to GMMA (Figure 2). This result is consistent with the lack of impact on the serum IgG response to Salmonella OAg (Figure 1F), as already observed with malaria antigens (Figure 1D,E).

### 3.2. GMMA as an Effective Carrier for Polysaccharides

Having verified that GMMA used as a carrier for protein antigen generated a significant enhanced immunogenicity for the antigen, we asked whether GMMA could be an effective carrier for polysaccharides. 

**Figure 3:**
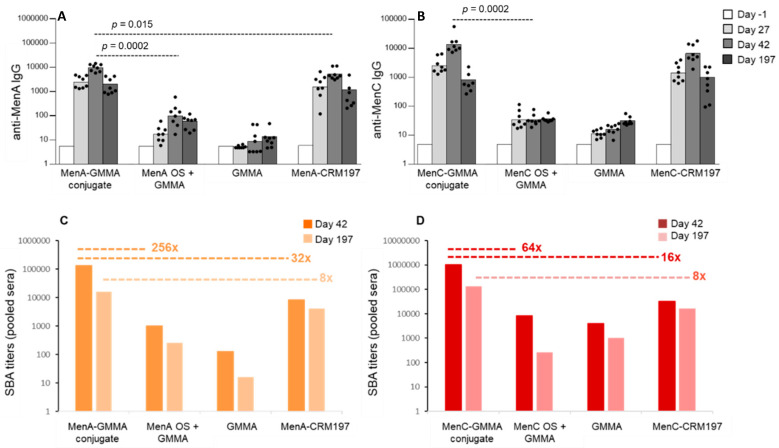
GMMA as a carrier for polysaccharides compared to traditional CRM197 conjugates. Eight CD1 mice per group were i.m. immunized at days 0 and 28, with MenA-GMMA (MenB) (**A**,**C**) or MenC-GMMA (MenB) conjugates (**B**,**D**) and their corresponding CRM197 conjugates at 1 µg Men oligosaccharide per dose, in the presence of Alhydrogel. MenB GMMA alone and their physical mixture with Men oligosaccharides were used as controls. Sera were collected at days 1, 27, 42 and 197 and analyzed for anti-PS-specific IgG response (**A**,**B**). Summary graphs of anti-PS IgG geometric mean units (bars) and individual antibody levels (dots) are reported. Anti-PS IgG were analyzed using a two-tailed nonparametric Mann–Whitney test. *p*-values (rounded to the nearest larger number) less than 0.05 were considered statistically significant. SBA titers of pooled sera collected 2 weeks and 6 months after second injection against MenA (**C**) or MenC (**D**) strains are reported.

MenC and MenA oligosaccharides were conjugated to MenB GMMA. To evaluate the capacity of saccharide-GMMA conjugates to elicit an anti-saccharide humoral immune response, we compared the saccharide-specific antibody response induced in mice by saccharide-GMMA conjugates, the corresponding physical mixtures of saccharide and GMMA or by CRM197 glycoconjugates as gold standards. In the presence of Alhydrogel, both saccharide-GMMA conjugates induced anti-saccharide-specific serum IgG levels comparable to corresponding CRM197 glycoconjugates, whereas the physical mixtures of GMMA and saccharides were poorly or non-immunogenic (Figure 3A,B). We also measured the capacity of sera derived from MenA- or MenC-immunized mice to promote the rabbit complement-mediated lysis of *Neisseria meningitidis*, by using a previously described in vitro bactericidal assay [30,41,42]. Immunization with saccharide-GMMA conjugates resulted in a stronger functional response (Figure 3C,D) compared to CRM197 glycoconjugates. Indeed, two weeks after the last immunization, the SBA measured in mice immunized with MenA- or MenC-GMMA conjugates was much higher than that induced by corresponding CRM197 glycoconjugates. The same result was not obtained by simply physically mixing oligosaccharides and GMMA, even if SBA titers against MenA and MenC strains were measurable, due to the immune response induced by GMMA per se. We also investigated persistence of the response induced and found that SBA titers remained higher in mice immunized with GMMA conjugates compared to CRM197 conjugates six months after the last immunization, despite a decrease in activity (Figure 3C,D). 

To understand whether the results obtained with MenA and MenC oligosaccharides were independent from the species involved in the GMMA production, we generated similar results using *S*. Typhimurium GMMA as a carrier. The data further confirmed the generally superior carrier effect of GMMA compared to CRM197 (Figure S2). 

**Figure 4:**
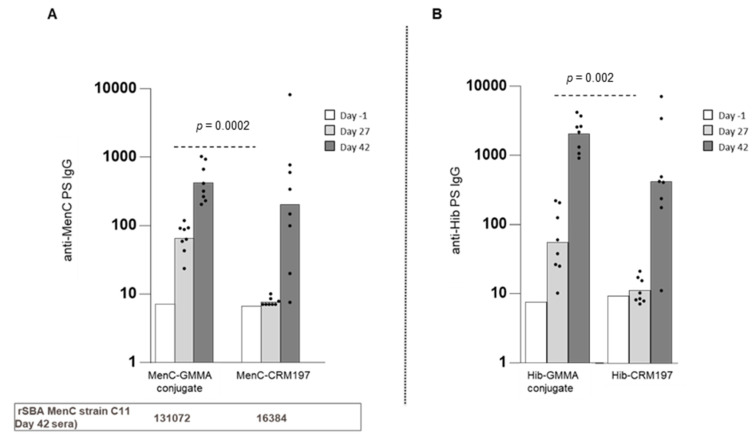
GMMA as a carrier for polysaccharides, formulations tested in the absence of Alhydrogel. Eight CD1 mice per group were s.c. immunized at days 0 and 28, with MenC-GMMA (MenB) or MenC-CRM197 conjugates ((**A**): 1 µg MenC oligosaccharide per dose). 8 adult rats per group were i.m. immunized at days 0 and 28, with Hib-GMMA (MenB) or Hib-CRM197 conjugates ((**B**): 0.5 µg Hib oligosaccharide per dose). Sera were collected at days 1, 27 and 42 and analyzed for anti-PS-specific IgG response. Summary graphs of anti-PS IgG geometric mean units (bars) and individual antibody levels (dots) are reported. SBA titers of pooled sera collected at day 42 from each group against MenC strain are reported in the table in panel A. Datasets were analyzed using a two-tailed nonparametric Mann–Whitney test. *p*-values (rounded to the nearest larger number) less than 0.05 were considered statistically significant.

When administered in absence of Alhydrogel, unlike the response elicited by the CRM197 glycoconjugate, MenC-GMMA (MenB) conjugate generated an anti-saccharide-specific IgG response after a single injection. Similar results were obtained by immunizing mice with Hib oligosaccharides conjugated to MenB GMMA (Figure 4). The superior functionality of saccharide-specific antibody response generated when immunizing with MenC-GMMA conjugate was also observed in absence of Alhydrogel formulation (Figure 4).

### 3.3. Chemical Linkage of Two Different Proteins or Saccharides on the Same GMMA Did Not Generate Immunointerference

**Figure 5:**
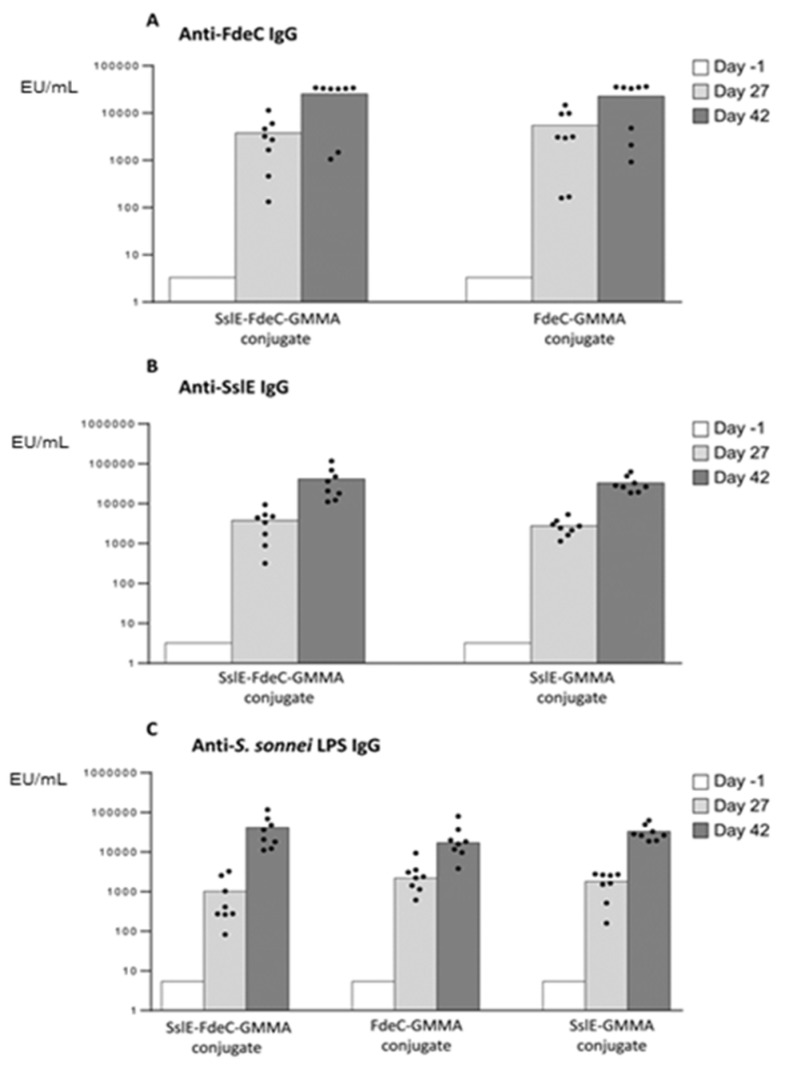
SslE and FdeC presented on the same *S. sonnei* GMMA particle compared to corresponding monovalent conjugates. Eight CD1 mice per group were i.m. immunized at days 0 and 28, with 5 µg total protein, in the presence of Alhydrogel. Sera were collected at days 1, 27 and 42 and analyzed for anti-*E. coli* antigen-specific (**A**,**B**) and anti-*S. sonnei* LPS (**C**) IgG response. Summary graphs of IgG geometric mean units (bars) and individual antibody levels (dots) are reported. Datasets were analyzed using a two-tailed nonparametric Mann–Whitney test. *p*-values (rounded to the nearest larger number) less than 0.05 were considered statistically significant.

Through chemical conjugation, we can also display multiple antigens on the same GMMA particle. We verified this by linking two different *E. coli* antigens, SslE [43,44] and FdeC [45], on the same *S. sonnei* GMMA particle [4], with the potential to protect against two major diarrheagenic pathogens, Enterotoxigenic *E. coli* (ETEC) and Shigella [46]. The conjugate presenting SslE and FdeC on the same GMMA particle (Table S1) induced anti-*E. coli* antigen-specific IgG response and anti-*S. sonnei* LPS IgG response, not significantly different from the anti-antigen-specific IgG response induced by corresponding monovalent conjugates (Figure 5). 

**Figure 6:**
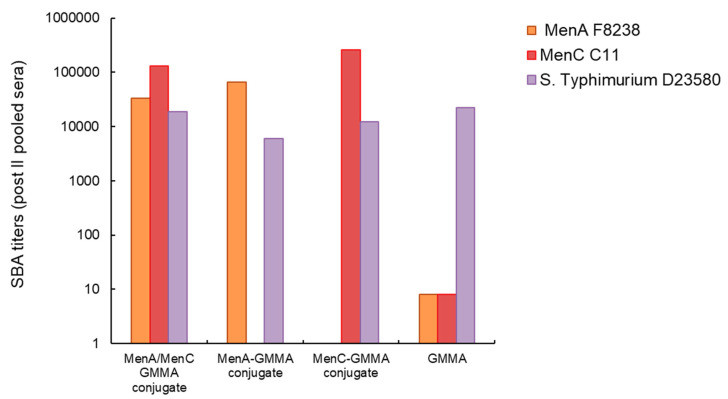
MenA and MenC oligosaccharides presented on the same *S*. Typhimurium GMMA particles compared to corresponding MenA or MenC monovalent GMMA conjugates and GMMA alone. Eight CD1 mice per group were i.m. immunized at days 0 and 28, with 1 µg MenA and MenC oligosaccharides per dose, in the presence of Alhydrogel. SBA titers of pooled sera collected 2 weeks after the second injection against MenA, MenC and *S*. Typhimurium strains are reported.

We also linked similar amounts of MenA and MenC oligosaccharides (Table S1) on *S.* Typhimurium GMMA. Also, in this case, no immune interference was detected by linking the two different saccharides on the same GMMA: the resulting conjugate elicited antibodies with strong bactericidal titers against MenA and MenC strains, similar to that induced by immunization with the monovalent MenA or MenC-GMMA conjugates (Figure 6). Again, the functionality of antibody response to *S.* Typhimurium was not negatively affected (Figure 6).

## 4. Discussion

There is still a huge global burden of infectious diseases, causing 91% of deaths in low- and middle-income countries [47], where implementation of existing vaccines is also difficult [15]. In the absence of commercial incentives, simple affordable technologies to target multiple diseases represent a preferred route for vaccine development. Use of technology platforms appear also beneficial to accelerate design, development and implementation of vaccines to fight antimicrobial resistance [48]. GMMA is a simple and effective platform to fight bacterial pathogens. Preclinical and clinical data have confirmed the potential of GMMA to induce strong immune responses [4,5,8,9,10,49,50]. Here, we have documented the use of GMMA as a “carrier” for antigens from other pathogens, as a general way to design multivalent combination vaccines against serious infectious diseases.

Expression of protein or saccharide antigens on OMV surface induces a strong antigen-specific immune response [3,51]. Recently, nanoparticle systems, including virus-like particles, gold nanoparticles and liposomes, have been explored for the display of carbohydrate and protein antigens, combining the multivalent presentation with the special physico-chemical properties of nano-sized particles [52,53,54]. Outer membrane protein complex (OMPC) from *Neisseria meningitidis* has been used as a carrier in the licensed *Haemophilus influenzae* type b conjugate vaccine [55,56] and also as a carrier for protein antigens, such as Pfs25H and Pfs230 malaria antigens, whose linking to OMPC resulted in an increased immunogenicity in mice compared to Pfs25H alone or physically mixed to OMPC and Pfs230-EPA, respectively [11,57].

Here, we selected chemical conjugation to link different types of antigen, even derived from phylogenetically distant pathogens, to GMMA surface. Chemical conjugation allows to play with antigens’ density, length and attachment site, facilitating the design of optimal vaccines. We are currently investigating these aspects. Once the optimal design for a specific vaccine combination has been identified, genetic tools could be used for antigen expression on GMMA surface, further reducing costs for vaccine manufacture. 

In the cases investigated, GMMA significantly improved the antigen-specific humoral immune response. When immunizing with GMMA-antigen conjugates, compared to antigen alone or physically mixed with GMMA, the antigen-specific IgG production was prevalently enhanced, but more interestingly, the functionality of the antibody was generally increased. Thus, GMMA used as an antigen carrier may generate a more effective immunization. Consistently, in our studies, we found immunization with GMMA-antigen conjugates as the best treatment to obtain a good antibody response after one administration. Also, by working with saccharide antigens, we demonstrated that the ability of GMMA conjugates to induce total polysaccharide-specific IgG titers was not inferior to more traditional CRM197 conjugates, but with stronger functionality, confirming what was recently found for nontyphoidal *Salmonella* GMMA compared with established glycoconjugates [49].

No enhanced immunogenicity was seen in mixtures of antigen and GMMA compared to antigen alone, the antigen needs to be displayed on GMMA surface to result in increased immunogenicity. This suggests that GMMA are not simply acting as an adjuvant. We believe that the immunogenicity of GMMA used as an antigen “carrier” could be the result of efficient antigen presentation to antigen-specific lymphocytes. Certainly, the mode of action of this effect of GMMA used as an antigen carrier is worthy of deep investigation because immunological mechanisms might be unveiled that could be exploited to also design more effective GMMA-based combination vaccines. Studies are ongoing to investigate the ability of GMMA to induce antigen-specific T-cell responses and the proportion of memory T-helper cells.

The generation of an effective immunization after one injection is remarkable. Indeed, reducing the number of injections would further decrease the cost of vaccination campaigns as well as improve the affordability of GMMA-based vaccines. In addition, less injections would facilitate the acceptability of the vaccine and the completion of vaccination schedules, consequently improving the immunization coverage. Finally, reducing the number of injections would minimize the adverse events following vaccination due to immunization errors, which is not a trivial question in large immunization campaigns. All these aspects are critical for immunization campaigns in low-income countries.

## 5. Conclusions

GMMA are characterized by a simpler manufacturing process compared to other traditional carriers [1,4] and our results demonstrate that GMMA can be used as a carrier for heterologous antigens to improve the immune response to the antigen without affecting the immune response to GMMA. This means that GMMA can play a dual role of antigen and carrier [58], facilitating the development of vaccines that are socially justified but that will require an innovative business case to be sustainable [15]. Importantly, we have shown that different antigens can be linked to the same GMMA particle with no immune interference. These features support the use of the GMMA technology for the development of simple multivalent vaccines, covering multiple diseases at the same time. By using GMMA and antigens (both proteins and polysaccharides) from different pathogens as models, we have demonstrated the broad applicability of this technology. 

This technology could be extended to other molecules, including viral proteins and small molecules, and represent a rapid tool to test the potentiality of the GMMA platform beyond its application to Gram-negative bacterial pathogens.

Based on the WHO recommendation to promote innovation in vaccine development to satisfy critical medical needs [18], our preclinical studies offer a proof of concept that GMMA exploited as a carrier might be a successful “plug and play” technology to design effective and affordable multivalent combination vaccines targeting different bugs at the same time.

## Supplementary Materials

The following are available online at https://www.mdpi.com/2076-393X/8/3/540/s1, Figure S1: GMMA conjugates formation is verified by SDS-PAGE/Western blot analysis. Figure S2: MenA and MenC oligosaccharides were conjugated to *S.* Typhimurium GMMA and compared in mice to corresponding MenA- or MenC-CRM197 conjugates, GMMA alone or physically mixed to Men oligosaccharides. Table S1: Conjugation conditions used and main characteristics of the GMMA conjugates tested in this study.
