# Supplementary material for: GMMA Is a Versatile Platform to Design Effective Multivalent Combination Vaccines
Source: Vaccines (Basel). 2020 Sep 17;8(3):540. doi: 10.3390/vaccines8030540 (PMC7564227; doi:10.3390/vaccines8030540)
Supplement: Supplementary file 1 [file vaccines-08-00540-s001.pdf]

vaccines

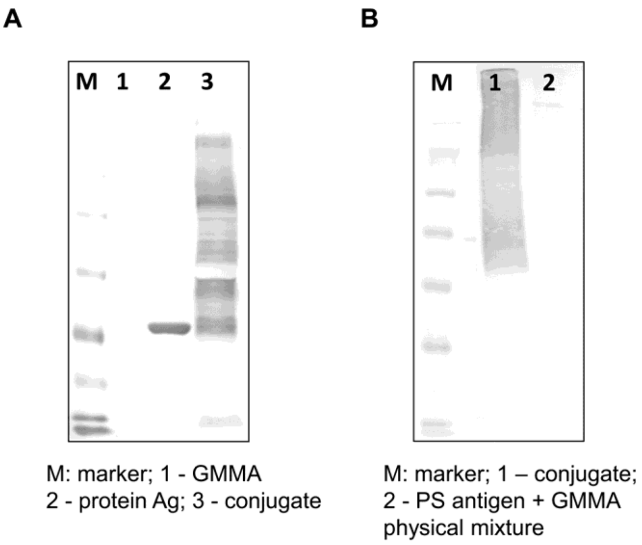

Figure S1. GMMA conjugates formation is verified by SDS PAGE/western blot analysis**.** Two examples are provided for conjugation of a protein antigen (**A**) and a polysaccharide antigen (B).

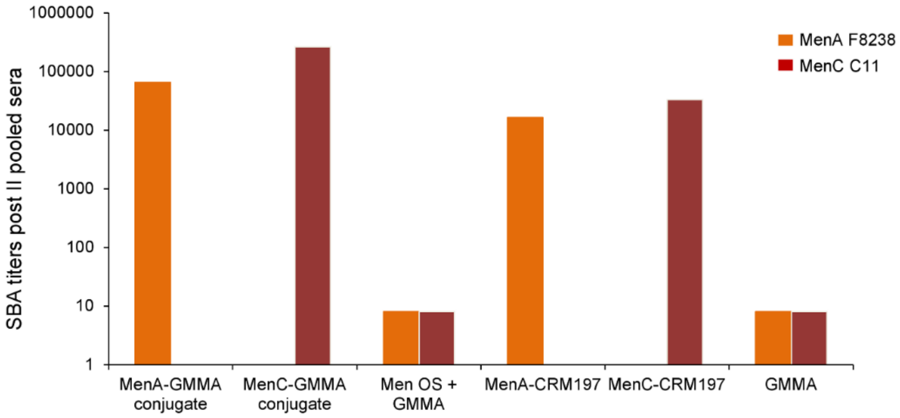

Figure S2. MenA and MenC oligosaccharides were conjugated to *S.* Typhimurium GMMA and compared in mice to corresponding MenA- or MenC-CRM197 conjugates, GMMA alone or physically mixed to Men oligosaccharides. Eight CD1 mice per group were i.m. immunized at days 0 and 28, with 1 µg Men oligosaccharide per dose, in the presence of Alhydrogel. SBA titers of pooled sera collected 2 weeks after second injection against MenA and MenC strains are reported.

Table S1. Conjugation conditions used and main characteristics of the GMMA conjugates tested in this study.

Conjugate	Chemistry	Targeting on GMMA	Conjugation Conditions	Antigen/GMMA w/w Ratio in Purified Conjugate	Number Saccharide Chains/GMMA Particle
STm GMMA-CSP	SH-maleimido	Proteins	GMMA/protein w/w ratio of 1:1; [GMMA] 96.7 ug/mL, pH 6, 4h, RT	0.33	na
STm GMMA-Pfs25	SH-maleimido	Proteins	GMMA/protein w/w ratio of 1:1; [GMMA] 285 g/mL, pH 5, 4h, RT	0.17	na
STm GMMA-fHbp	SH-maleimido	Proteins	GMMA/protein w/w ratio of 2:1; [GMMA] 4 mg/mL, pH 6, 5h, RT	0.3	na
S. sonnei GMMA-SslE	Reductive amination	LPS	GMMA/protein w/w ratio of 1:1; [GMMA] 1.23 mg/mL, pH 6.5, ON, RT	0.35	na
S. sonnei GMMA-FdeC	BS3	Proteins	GMMA/protein w/w ratio of 1:1; [GMMA] 5.75 mg/mL, pH 7.4, ON, RT	0.12	na
Bivalent *S. sonnei* GMMA-SslE-FdeC	BS3 for FdEc and reductive amination for SslE	Both proteins and LPS	BS3: GMMA/protein w/w ratio of 1:1; [GMMA] 6.45 mg/mL, pH 7.4, ON, RT<br>Red am: GMMA/protein w/w ratio of 1:1; [GMMA] 1.18 mg/mL, pH 6.5, ON, RT	0.08 FdeC, 0.39 SslE	na
MenA-(MenB)GMMA	SIDEA	Proteins	GMMA/OS w/w ratio of 1:10; [GMMA] 10 mg/mL, pH 7.2, ON, RT	0.054	2490
MenC-(MenB)GMMA	SIDEA	Proteins	GMMA/OS w/w ratio of 1:10; [GMMA] 10 mg/mL, pH 7.2, ON, RT	0.10	4523
Bivalent MenAC-(MenB)GMMA	SIDEA	Proteins	GMMA/MenA OS/MenC OS w/w/w ratio of 1:10:10; [GMMA] 10 mg/mL, pH 7.2, ON, RT	0.103 (MenA)<br>0.12 (MenC)	3847 (MenA),<br>5168 (MenC)

Conjugate	Chemistry	Targeting on GMMA	Conjugation Conditions	Antigen/GMMA w/w Ratio in Purified Conjugate	Number Saccharide Chains/GMMA Particle
MenA-(STm)GMMA	SIDEA	Proteins	GMMA/OS w/w ratio of 1:10; [GMMA] 10 mg/mL, pH 7.2, ON, RT	0.032	417
MenC-(STm)GMMA	SIDEA	Proteins	GMMA/OS w/w ratio of 1:10; [GMMA] 10 mg/mL, pH 7.2, ON, RT	0.038	479
Bivalent MenAC-(STm)GMMA	SIDEA	Proteins	GMMA/MenA OS/MenC OS w/w/w ratio of 1:10:10; [GMMA] 10 mg/mL, pH 7.2, ON, RT	0.029 (MenA) 0.036 (MenC)	378 (MenA), 454 (MenC)
Hib-(MenB)GMMA	SIDEA	Proteins	GMMA/OS w/w ratio of 1:3; [GMMA] 10.5 mg/mL, pH 7.2, ON, RT	0.081	4230
